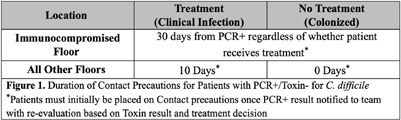# Appropriate De-Escalation of Contact Precautions in Patients with Discordant C. difficile PCR+/Toxin- Testing

**DOI:** 10.1017/ash.2025.402

**Published:** 2025-09-24

**Authors:** J. Hunter Fraker, Matthew Lee

**Affiliations:** 1 Beth Israel Deaconess Medical Center;Preeti Mehrotra, Bidmc; 2 Beth Israel Deaconess Medical Center

## Abstract

**Background:** Contact precautions reduce nosocomial spread of Clostridioides difficile (C. difficile). However, they can decrease patient interactions with providers and delay discharges, so it is imperative precautions are discontinued when appropriate. Patients with discordant C. difficile testing (PCR+/Toxin-) require clinical judgment to determine infection versus colonization. Our institution’s C. difficile isolation protocol categorizes duration based on C. difficile treatment and type of patient floor to reflect this. We transitioned to a new electronic medical record (EMR) in June 2024, which included additional decision support for Contact precaution discontinuation. Prior to new EMR implementation, we hypothesized that patients with discordant C. difficile testing were not being appropriately de-escalated from precautions despite meeting institutional criteria. **Methods:** This was a retrospective chart review of inpatients admitted to our hospital who had discordant C. difficile testing (PCR+/Toxin-) from July 1, 2023 to October 10, 2023. Patients were excluded if there was no indication of PCR+ (critical value) notification to providers or if patients were on Contact precautions with an additional indication to C. difficile. The primary outcome was the proportion of patients with discordant C. difficile testing who had Contact precautions appropriately discontinued based on internal criteria (Figure 1). **Results:** A total of 90 patients had discordant C. difficile testing during the study period; 10 were excluded. In the study cohort (n=80), 33.8% (27/80) did not have orders placed for Contact precautions at any time despite positive PCR (Figure 2). Of the remaining 53 patients who were placed on Contact precautions, the median start time of Contact precautions after PCR notification was one hour and 20 minutes.

Of patients who were placed on Contact precautions (n=53), 30.2% (16/53) were treated and deemed to have clinical infection, while 69.8% (37/53) were diagnosed with colonization and not treated for C. difficile infection. Overall, 84.9% (45/53) had appropriate de-escalation of Contact precautions; the remaining 8 (15.1%) had inappropriate de-escalation of Contact precautions and were all in the not treated/colonized group. **Conclusion:** In our single-institution study, we found higher than expected rates of appropriate Contact precaution initiation and discontinuation; however, 15% still had inappropriate precaution durations. Coupled with the surprising number of patients not initiated on precautions at any time after positive PCR, our results highlighted the need to build clear clinical decision support tools with our new EMR and continual surveillance of providers for adherence to isolation protocols post-implementation.

**Figure:**
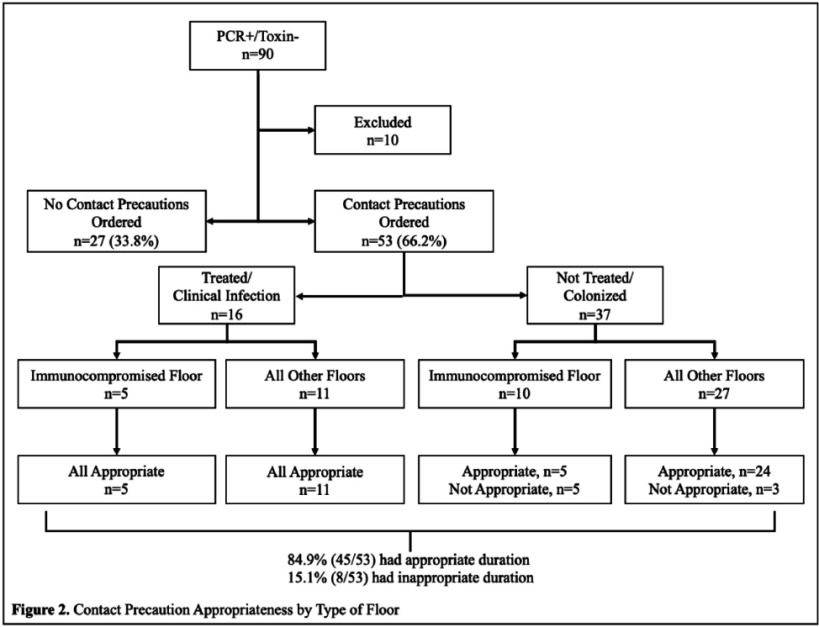

**Figure:**